# Age-associated deficient recruitment of 53BP1 in G1 cells directs DNA double-strand break repair to BRCA1/CtIP-mediated DNA-end resection

**DOI:** 10.18632/aging.202419

**Published:** 2020-12-27

**Authors:** Teresa Anglada, Anna Genescà, Marta Martín

**Affiliations:** 1 Department of Cell Biology, Physiology and Immunology, Universitat Autònoma de Barcelona, Bellaterra 08193, Spain

**Keywords:** aging, double-strand break repair, 53BP1, BRCA1, non-homologous end-joining

## Abstract

DNA repair mechanisms play a crucial role in maintaining genome integrity. However, the increased frequency of DNA double-strand breaks (DSBs) and genome rearrangements in aged individuals suggests an age-associated DNA repair deficiency. Previous work from our group revealed a delayed firing of the DNA damage response in human mammary epithelial cells (HMECs) from aged donors. We now report a decreased activity of the main DSB repair pathways, the canonical non-homologous end-joining (c-NHEJ) and the homologous recombination (HR) in these HMECs from older individuals. We describe here a deficient recruitment of 53BP1 to DSB sites in G1 cells, probably influenced by an altered epigenetic regulation. 53BP1 absence at some DSBs is responsible for the age-associated DNA repair defect, as it permits the ectopic formation of BRCA1 foci while still in the G1 phase. CtIP and RPA foci are also formed in G1 cells from aged donors, but RAD51 is not recruited, thus indicating that extensive DNA-end resection occurs in these breaks although HR is not triggered. These results suggest an age-associated switch of DSB repair from canonical to highly mutagenic alternative mechanisms that promote the formation of genome rearrangements, a source of genome instability that might contribute to the aging process.

## INTRODUCTION

Aging is a complex process that implies the loss of physiological integrity and affects the DNA of cells. Several studies have reported increased frequencies of both DNA double-strand breaks (DSBs) and genome rearrangements in different tissues of aged organisms and senescent cells [1, 2]. Also, mutations in some DSB repair genes have been described as causing a premature aging phenotype [3], thus pointing to a relationship between defective DNA repair and age. In this regard, the direct measurement of DSB repair events using plasmid constructs has demonstrated an age-associated decrease in the efficiency and fidelity of the repair function in mice, rats and human cells [4–6]. Thus, several evidences link aging with a decline in DSB repair efficiency that could account for the accumulation of unrepaired DNA damage and increased genome instability with age. However, the nature of the DSB repair defect underlying the age-related accumulation of DNA damage remains an open question.

There are two main DSB repair pathways operating in mammalian cells: the canonical non-homologous end-joining (c-NHEJ), which is able to join scarcely resected broken DNA ends that share little or no homology, and the homologous recombination (HR) pathway, which needs extensive DNA resection and uses the sister chromatid as a template for repair. The choice of either DSB repair pathway is highly regulated, cell cycle being a major determinant [7–9]. While the c-NHEJ is active throughout the cycle and is the predominant pathway during G1, HR activates from mid-S to G2, when a sister chromatid is available as a template for repair.

At a molecular level, the balance between 53BP1 and BRCA1 plays an important role in the DSB repair pathway choice by modulating end resection [10–12]. After DNA damage induction, multiple sites of 53BP1 are phosphorylated by the ataxia-telangiectasia mutated (ATM) kinase and 53BP1 is rapidly relocated to the break site [13]. Phosphorylated 53BP1 directly recognizes the ubiquitination of H2A at lysine 15 (H2AK15ub) [14], but the selective recognition of H4 di-methylation at lysine 20 (H4K20me2) is also required for the focal accumulation of 53BP1 at DSBs [15]. This epigenetic mark is catalyzed by methyltransferases SETD8 and SUV4-20 and >80% of nucleosomes in G1 cells present H4K20me2 [16]. Also, 53BP1 binding affinity with H4K20me2 is enhanced by the deacetylation of H4 at lysine 16 [17, 18]. Once 53BP1 accumulates at the DSBs it serves as a platform for the recruitment of other factors that direct repair to the c-NHEJ, while restricting BRCA1 accumulation at the DSB during the G1 phase of the cell cycle [10].

Progression into S phase enables BRCA1 recruitment to DSBs by recognition of the unmethylated lysine 20 of histone H4 (H4K20me0) on newly synthesized DNA [19]. BRCA1 interacts with CtIP in a cyclin-dependent kinase (CDK)-dependent manner and promotes DNA-end resection, thus inhibiting c-NHEJ [20, 21]. The initial steps of resection are directed by the CtIP/MRE11 complex [22], which, along with other nucleases, generate long stretches of single stranded DNA (ssDNA) that are coated by RPA [23]. RAD51 is a core protein of the HR pathway that progressively displaces the RPA from ssDNA to form a nucleoprotein filament that is competent to invade a sister chromatid strand and use it as a template for repair [24]. Finally, DNA ends are ligated and the original sequence is restored without error.

In addition, under specific cellular conditions, alternative highly mutagenic pathways, such as the alternative end-joining (Alt-EJ), can repair those DSBs that have suffered from DNA-end resection. This mechanism promotes ligation of resected DNA ends, thus increasing the probability of introducing alterations in the DNA sequence [25]. In the last few years, there have been great advances in describing the mechanisms that regulate the interplay between the different DSB repair pathways (c-NHEJ, HR and Alt-EJ) and their components. However, the decline in the efficiency of these pathways with aging, and the factors that eventually influence the interplay between DNA repair pathways in this context, remain to be explored.

In a previous work from our group, we measured the frequency of γH2AX foci in G1 human mammary epithelial cells (HMECs) from young and aged donors after exposure to γ-rays [26]. Results of that work showed that an age-associated delay in the firing of the DNA damage response (DDR) was responsible for the repair deficiency observed in HMECs from older women [26]. In order to find out the exact nature of this repair defect, in the present work we evaluated the activity of the c-NHEJ and HR repair pathways and the dynamics of the main proteins involved in these mechanisms after DSB induction. We observed a deficiency in the recruitment of 53BP1 to DSBs in cells from aged donors. Absence of 53BP1 from some DSBs in G1 cells allows ectopic access of BRCA1, CtIP and RPA but not RAD51 to these breaks. We propose that increasing age hampers proper recruitment of 53BP1 to G1 DSBs. This defect is probably influenced by changes in the epigenetic landscape affecting the aging genome, such as H4K20 methylation, directly involved in the recruitment of 53BP1. DSBs devoid of 53BP1 are accessible to the DNA-end resection machinery, thus switching DSB repair from c-NHEJ to error-prone mechanisms such as Alt-EJ, which could contribute to the reported age-associated formation of genomic rearrangements.

## RESULTS

### The efficiency of the c-NHEJ and HR DSB repair pathways decreases with age

In a previous study, we described an age-associated increase in the frequency of DNA DSBs in HMECs following ionizing radiation (IR) exposure due to an age-related delayed firing of the DDR [26]. To investigate the nature of this repair defect, here we used HMECs derived from mammary tissues of female donors classified as young donors (YDs ≤ 27 years old) and aged donors (ADs ≥ 60 years old). Low population doubling (PD) cells’ (< 20 PD) were used in all of the experiments to rule out any effect of replicative cellular senescence on DNA repair.

**Figure 1:**
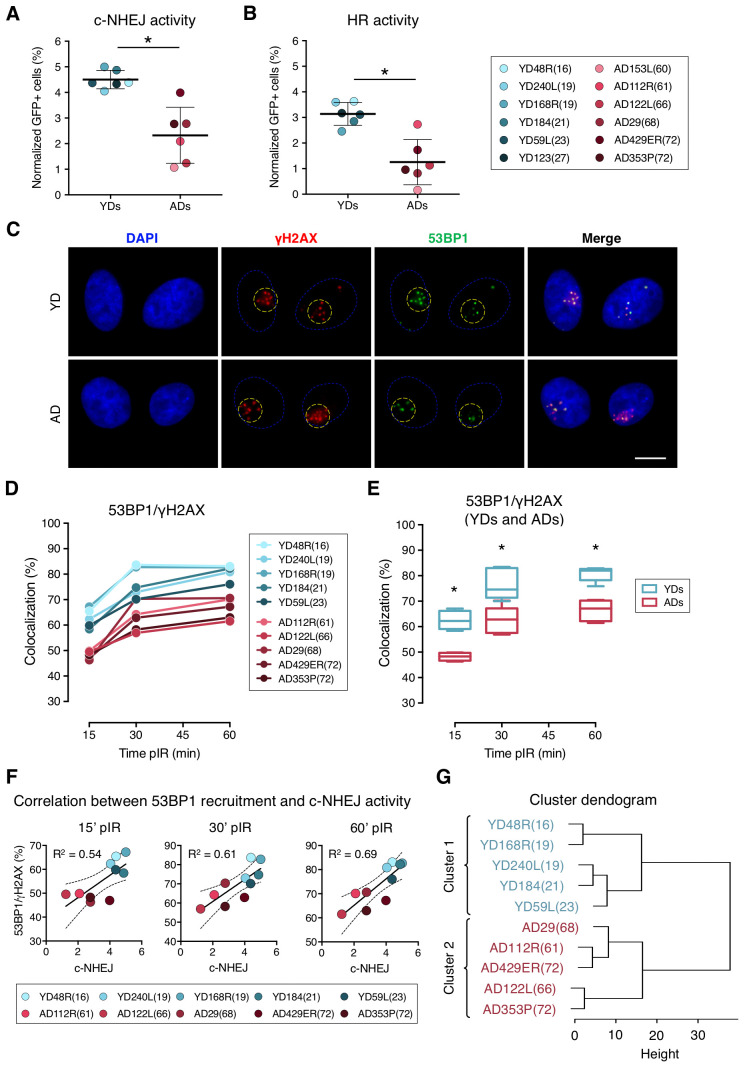
**Decreased efficiency of DNA–DSB repair and deficient recruitment of 53BP1 in aged donor cells.** (**A**, **B**) Normalized frequency of GFP-positive cells after co-transfection with pimEJ5GFP (**A**) or pDRGFP (**B**) and the I-SceI expressing plasmids. Mean and SD are indicated (* *p* < .001; *t*-test). (**C**) Immunofluorescent labeling of cell nuclei (DAPI, blue), γH2AX (Cy3, red) and 53BP1 (A488, green). γH2AX and 53BP1 foci were scored within the irradiated pore area (yellow dotted lines). Scale bar = 10 μm. (**D**, **E**) Percentage of 53BP1/γH2AX foci colocalization for five young and five aged donors (**D**) and summary values for each age group (**E**). Boxes include data from the upper to the lower quartile and whiskers compile minimum to maximum values (* *p* < .0001; *n* is stated in Supplemental Table 2; two-way ANOVA + Bonferroni). (**F**) Correlation between c-NHEJ efficiency and 53BP1/γH2AX foci colocalization. Best-fit line, 95% confidence bands (dotted lines) and Pearson’s correlation coefficient (R^2^) are indicated (*p* < .05). (**G**) Hierarchical clustering of the ten donors according to the percentage of 53BP1/γH2AX foci colocalization.

First, we evaluated the efficiency of the c-NHEJ and HR pathways by transfecting the cells with reporter plasmids. Transfection efficiency values for each donor were calculated using a constitutively GFP-expressing plasmid. As expected for primary cells, these values were low, but there were no statistical differences between the two age groups (Supplementary Table 1; YDs = 9.18%; ADs = 11.51%, t-test; *p*-value > .05). Then we evaluated the efficiency of the c-NHEJ and HR pathways by transfecting the cells with the reporter plasmids pimEJ5GFP or pDRGFP together with an I-SceI enzyme-expressing plasmid (Supplementary Figure 1A–1C). ADs showed a statistically significant decrease in the c-NHEJ activity, as shown by the normalized frequency of GFP-positive cells (4.50% in YDs and 2.32% in ADs; *t*-test; *p*-value < .001) (Figure 1A and Supplementary Table 1). Consistent with this, c-NHEJ activity was negatively correlated with the residual number of γH2AX foci scored 24 h after exposure to 1 Gy of γ-rays in each donor in our previous study [26] (R^2^ = 0.39; *p*-value = 0.0308) (Supplementary Figure 1D). Therefore, the c-NHEJ repair pathway is less active with age and this defect most probably contributes to the accumulation of residual DSBs in ADs.

In contrast to the c-NHEJ pathway, the HR pathway only operates in S and G2 phases of the cell cycle. According to this, all donors showed less HR than c-NHEJ activity, as shown by the normalized frequencies of GFP-positive cells (Figure 1B; Supplementary Table 1). Our results showed that the HR repair pathway activity was also reduced in ADs compared to YDs (3.14% for YDs and 1.33% for ADs), and the difference was statistically significant (*t*-test; *p*-value < .001). A reduced activity of the HR repair pathway in ADs could be due to an increased frequency of ADs’ cells halted in the G1 phase. However, the cell cycle analysis showed no significant differences in the distribution of the cell cycle phases between YD and AD cells (two-way ANOVA and Bonferroni multiple correction test; *p*-value > .05) (Supplementary Figure 1F–1G). Finally, we observed that the donors with low HR activity also showed low c-NHEJ activity (R^2^ = 0.54, *p*-value = 0.0065) (Supplementary Figure 1E) and they mostly corresponded to aged donors. These results indicate that the age-related DSB repair defect is not restricted to a single pathway, but instead affects both main DSB repair pathways, thus contributing to the accumulation of DNA damage in aged organisms.

### Age-associated decrease in the recruitment of 53BP1 to radiation-induced DSBs

The DDR operates in a highly coordinated manner and protein recruitment dynamics are essential for the proper triggering of the DNA repair mechanisms. Thus, we next hypothesized that the repair defect observed in ADs could be due to an impaired recruitment of DNA damage signaling and repair proteins to DSB sites. The dynamics of recruitment of the main proteins responsible for the repair pathway choice, 53BP1 and BRCA1, were evaluated in cells from the two age groups. To this end, DSBs were induced in localized areas of cell nuclei using the micro-irradiation technique described by Suzuki et al. [27]. DSBs were identified at 15, 30 and 60 min post-irradiation (pIR) as discrete γH2AX foci. The newly induced DSBs in localized nuclear areas could be clearly differentiated from the basal ones (Figure 1C), whose frequency was low but significantly increased in aged donors [26].

We first checked the colocalization of 53BP1 and γH2AX at radiation-induced DSBs. To do so, micro-irradiated nuclear areas were localized, and individualized γH2AX foci were scored. Next, the coincidence in space of 53BP1 focus with each γH2AX foci site was visually determined (Figure 1C) and referred to as colocalization. The colocalization of 53BP1 with γH2AX foci increased with time after irradiation both in YDs and ADs, reaching a plateau at 60 min pIR (Figure 1D). However, percentages of colocalization were significantly lower for the ADs in comparison to the younger ones at all times analyzed (YDs: 62.59% at 15 min, 76.39% at 30 min and 81.00% at 60 min; ADs: 48.19% at 15 min, 62.42% at 30 min and 66.79% at 60 min; two-way ANOVA and Bonferroni multiple correction test; *p*-value < .0001) (Figure 1E; Supplementary Table 2).

An age-related impaired recruitment of 53BP1 to DSBs could account for the delayed firing of the DDR and the increased frequency of residual breaks previously reported in AD cells as well as for the reduced efficiency of the c-NHEJ pathway observed in the present study. Frequencies of 53BP1 recruitment to DSBs for each donor positively correlated with values of c-NHEJ efficiency evaluated with the plasmid reporter system at all time points analyzed (R^2^ = 0.54 at 15 min; R^2^ = 0.61 at 30 min and R^2^ = 0.69 at 60 min pIR) (Figure 1F). Also, 53BP1 recruitment to DSBs at 60 min pIR negatively correlated with the frequencies of γH2AX foci in HMECs from the same donors reported previously [26] both in non-irradiated samples (R^2^ = 0.76) and 24 h after irradiation (R^2^ = 0.79) (Supplementary Figure 2), indicating that proper recruitment of 53BP1 favors γH2AX foci clearance. Finally, data from the 53BP1/γH2AX colocalization at the different time points hierarchically clustered donors into two differentiated groups that coincided with chronological age (Figure 1G), revealing that the frequency of DSBs signaled by 53BP1 is a good marker of age. Altogether, these results indicate that 53BP1 recruitment to DSB sites soon after DNA damage induction is impaired in the HMECs from aged donors, and it translates into a lower activity of the c-NHEJ and into an increased frequency of both basal and induced γH2AX foci.

### BRCA1 is efficiently recruited to DSBs in G2 phase HMECs from older donors

**Figure 2:**
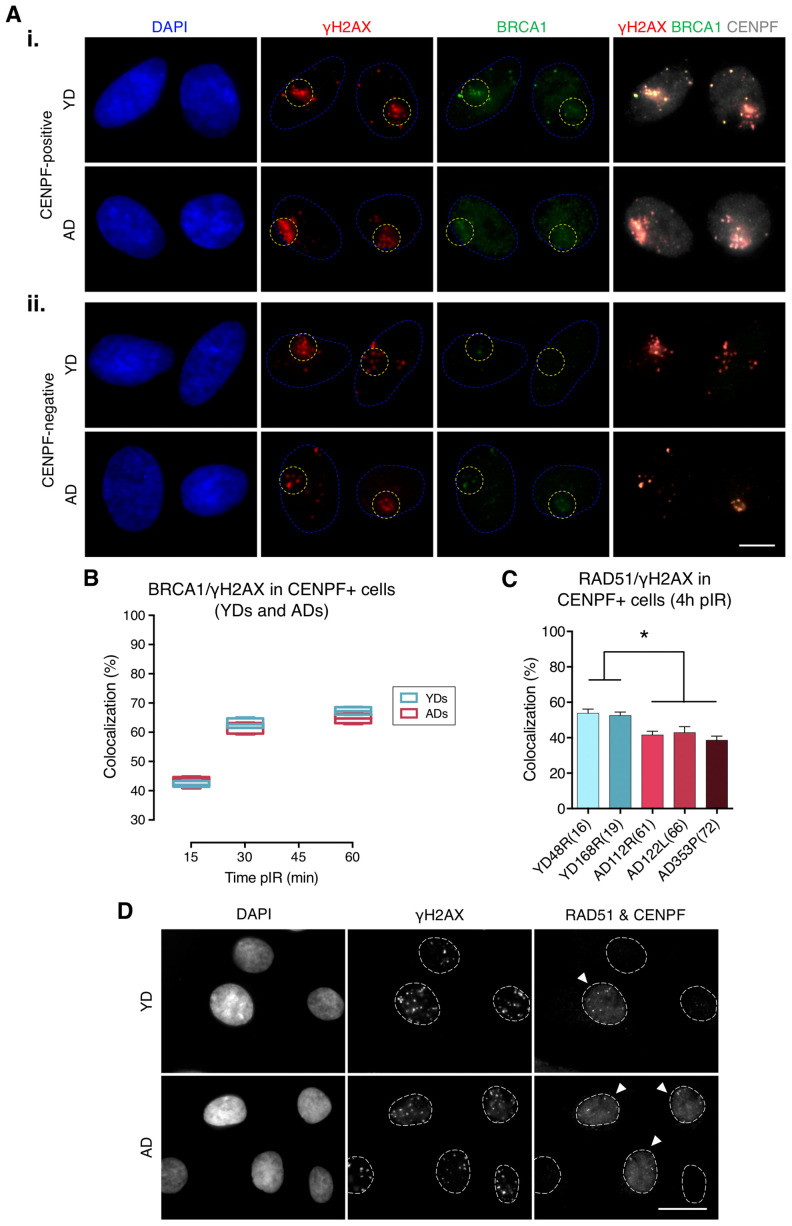
**Recruitment of BRCA1 to DNA damage sites in G2 is not impaired in cells from aged donors.** (**A**) Immunofluorescent labeling of cell nuclei (DAPI, blue), γH2AX (A594, red), BRCA1 (A488, green) and CENPF (A532, grey). γH2AX and BRCA1 foci were scored within the irradiated pore zone (yellow dotted lines) in CENPF-positive (i) or CENPF-negative (ii) cells. Scale bar = 10 μm. (**B**) Percentage of BRCA1/γH2AX foci colocalization in CENPF-positive HMECs from four YDs and four ADs. Boxes include data from the upper to the lower quartile and whiskers compile minimum to maximum values (*n* is stated in Supplementary Table 3). (**C**) Percentage of RAD51/γH2AX foci colocalization in CENPF-positive cells at 4 h after irradiation (5 Gy, γ-rays). Error bars indicate SEM (* *p* < .05; *n* ≥ 500 γH2AX foci/donor; one-way ANOVA + Tukey). (**D**) Immunofluorescent labeling of cell nuclei (DAPI), γH2AX (A488), RAD51 (A594) and CENPF nuclear staining (A532) at 4 h after exposure to 5 Gy of γ-rays. Arrowheads indicate G2 (CENPF-positive) cells. Scale bar = 20 μm.

Next, we assessed BRCA1 recruitment to radiation-induced DSBs by quantifying colocalization of BRCA1 with γH2AX foci in G2 cells identified by positive CENPF staining, a protein that progressively accumulates from the S phase to mitosis (Figure 2A, panel i). BRCA1 recruitment kinetics followed a pattern resembling that of 53BP1: it increased with time after micro-irradiation in both YDs and ADs, reaching a plateau at 60 min pIR. Nevertheless, there were no detectable differences in the percentage of BRCA1/γH2AX colocalization between the two age groups (YDs: 42.55% at 15 min, 62.67% at 30 min and 67.42% at 60 min; ADs: 43.04% at 15 min, 61.87% at 30 min and 64.84% at 60 min; two-way ANOVA and Bonferroni multiple correction test; *p*-value > .05) (Figure 2B; Supplementary Table 3). Given that BRCA1 recruitment to DSBs in G2 cells from ADs was as effective as in those from YDs, we ruled out an age-associated deficiency in BRCA1-mediated DSB recognition. However, difficulties related to the recruitment of other effector proteins downstream of BRCA1 could account for the reduced activity of the HR repair pathway in cells from older donors. Indeed, ADs showed reduced percentages of RAD51 colocalization with γH2AX foci in G2 cells at 4 h after exposure to 5 Gy of γ-rays (53.37% in YD vs. 40.91% in AD; one-way ANOVA and Tukey multiple correction test; *p*-value < .05) (Figure 2C, 2D). Altogether, the results suggest that an impaired or delayed RAD51 recruitment to DSBs could affect the proper progression of homologous recombination repair with age and account for the decreased efficiency of the HR pathway detected with the plasmid reporter assay in cells from older donors.

### Decreased mRNA levels of H4K20 methyltransferase SETD8 in HMECs from older donors

**Figure 3:**
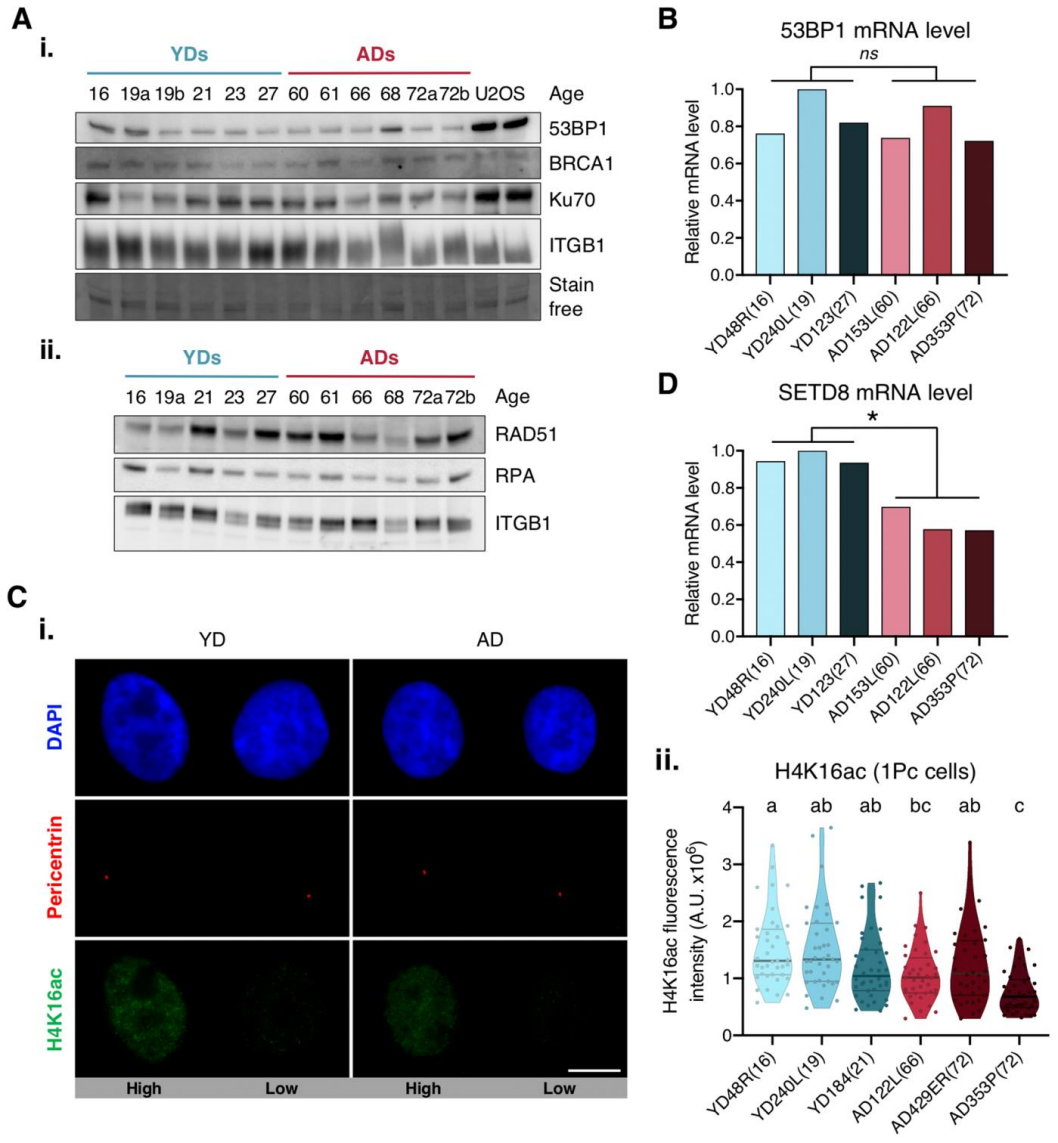
**Expression levels of 53BP1, SETD8 and H4K16ac.** (**A**) Western blot analysis of c-NHEJ and HR factors. Basal levels of (i) high and (ii) low molecular weight DNA repair proteins. Stain-free technology and/or Integrin β1 (ITGB1) have been used for sample normalization and U2OS cells were used as a positive control. (**B**, **D**) RT-qPCR analysis of 53BP1 (**B**) and SETD8 (**D**). GAPDH and β-actin were used as reference genes (* *p* < .05, *ns* *p* > .05; *t*-test). (**C**) H4K16ac analysis. (i) Immunofluorescent labeling of cell nuclei (DAPI, blue), H4K16ac (A488, green) and pericentrin (A594, red). Representative G1 cells with high or low H4K16ac fluorescence intensity are shown. Scale bar = 10 μm. (ii) Fluorescence intensity of H4K16 acetylation in G1 cells (1 perincentrin signal). Each dot corresponds to one cell and the mean and quartiles are indicated (a≠b≠c *p* < .05; *n* = 40 cells/donor; Kruskal–Wallis + Dunn).

We next aimed to explore the causes underlying deficient recruitment of repair proteins in HMECs from older individuals. Our first hypothesis was that protein expression was differentially regulated by age. Since the expression of DNA repair enzymes has been evaluated in cells from aged individuals and senescent cells with inconsistent results [6, 28], we measured 53BP1 gene expression by RT-qPCR and protein levels by Western blot in HMECs from old and young donors. Although some variation was detected amongst donors, no significant differences in 53BP1 mRNA and protein levels were observed between the two age groups (Figure 3A, panel i, and 3B). Likewise, Western blot results showed no age-related differences for 53BP1’s effector protein Ku70, or for other proteins directing repair to the HR, like BRCA1, RPA and RAD51. Again, the levels of these proteins showed inter-individual variations, but no age-associated tendency was detected (Figure 3A). Thus, we conclude that the decline in c-NHEJ and HR repair and the recruitment defect observed in ADs is not due to depletion of DNA repair proteins.

We next argued that epigenetic alterations associated with aging could be influencing 53BP1 recruitment efficiency in AD cells. The focal accumulation of 53PB1 on DSBs relies on the specific binding of 53BP1 to the H4K20me2 [29–31]. Conversely, lysine 16 acetylation of the same histone H4 (H4K16Ac) significantly reduces 53BP1 interaction with H4K20me2 [32, 33]. Hence, we measured H4K16 acetylation levels in G1 cells (only 1 pericentrin mark) from young and aged donors. Regardless of donor’s age, H4K16ac fluorescence intensity showed high cell-to-cell variability amongst cells of the same donor (Figure 3C), and no statistically significant difference between age groups was found (Figure 3C, panel ii). We next hypothesized that an inefficient methylation of H4K20 in cells from ADs could translate into a deficient accumulation of 53BP1 at DSBs. The first step to methylate H4K20 is the monomethylation by lysine methyltransferase SETD8 [34]. Decreased SETD8 mRNA levels have been reported in senescent cells and in *in vitro* aged fibroblasts [35, 36]. Thus, we measured the expression of SETD8 by RT-qPCR in HMECs from young and aged donors. Interestingly, results revealed a significant decrease in SETD8 mRNA levels in HMECs from aged donors in comparison to the younger ones (mean relative expression: YD = 0.96; AD = 0.62; t-test; *p*-value < .01) (Figure 3D). We speculate that the observed decrease of SETD8 could translate into an age-associated increase of H4K20me0 sites to which 53BP1 recruitment would be hindered, while occupancy by other DNA repair proteins, such as BRCA1, would be favored.

### In the absence of 53BP1, BRCA1 is ectopically recruited to DSBs in G1

**Figure 4:**
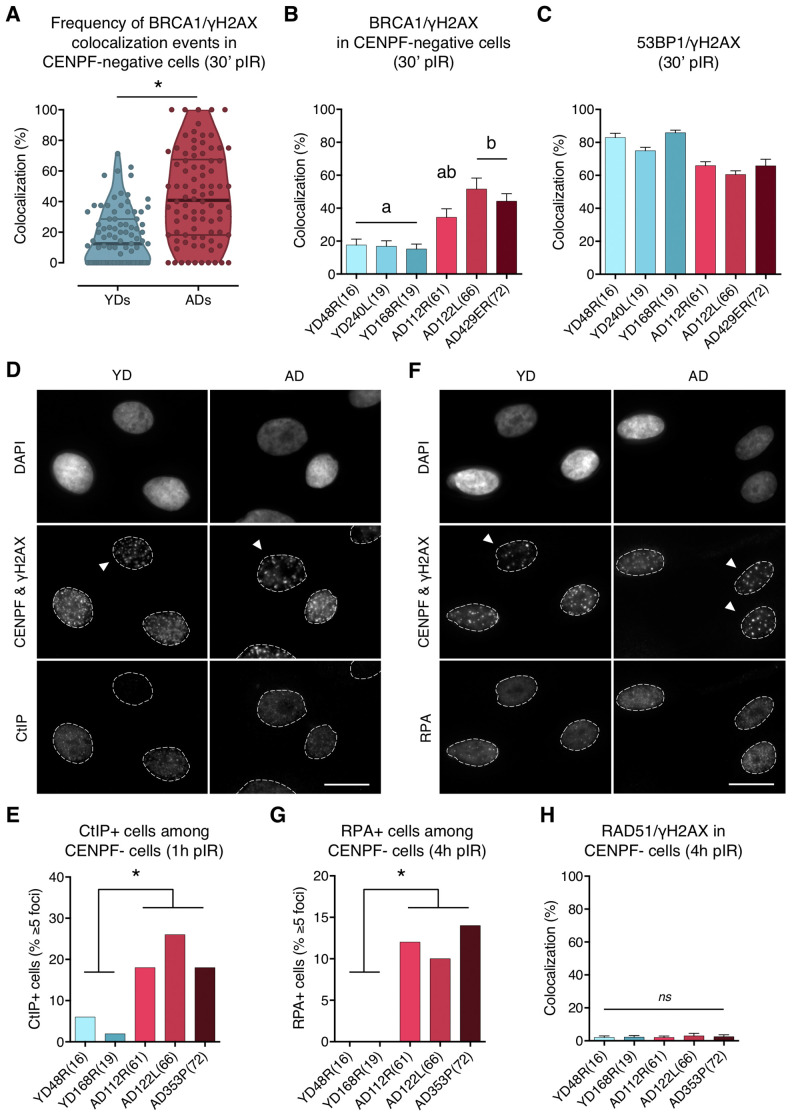
**BRCA1, CtIP and RPA but no RAD51 are ectopically recruited to DNA DSBs in G1 cells from aged donors.** (**A**) Percentage of BRCA1/γH2AX foci colocalization in CENPF-negative cells at 30 min after irradiation for each age group. Each dot corresponds to the fraction of BRCA1 and γH2AX foci colocalizing within one pore and the mean and quartiles are indicated (* *p* < .0001; *n* is stated in Supplementary Table 4; Mann–Whitney test). (**B**) Summary values for individual donors. Error bars indicate SEM (a≠b *p* < .05; *n* is stated in Supplementary Table 4; Kruskal–Wallis + Dunn). (**C**) Percentage of 53BP1/γH2AX foci colocalization for individual donors at 30 min after irradiation. Error bars indicate SEM (*n* is stated in Supplementary Table 2). (**D**) Immunofluorescent labeling of cell nuclei (DAPI), CENPF (A532), γH2AX (A594) and CtIP (A488). Arrowheads indicate G1 (CENPF-negative) cells. Scale bar = 20 μm. (**E**) Frequency of CtIP-positive HMECs (≥ 5 foci) at 1 h after irradiation (5 Gy, γ-rays). Analysis was restricted to CENPF-negative cells (* *p* < .05; *n* ≥ 50 cells/donor; Fisher’s exact test). (**F**) Immunofluorescent labeling of cell nuclei (DAPI), CENPF (A532), γH2AX (A594) and RPA (A488). Arrowheads indicate G1 (CENPF-negative) cells. Scale bar = 20 μm. (**G**) Frequency of RPA-positive HMECs (≥ 5 foci) at 4 h after irradiation (5 Gy, γ-rays). Analysis was restricted to CENPF-negative cells (* *p* < .05; *n* ≥ 50 cells/donor; Fisher’s exact test). (**H**) Percentage of RAD51/γH2AX foci colocalization in CENPF-negative cells at 4 h after irradiation (5 Gy, γ-rays). Error bars indicate SEM (*ns* *p* > .05; *n* ≥ 1000 γH2AX foci/donor; Kruskal–Wallis + Dunn).

In G1 cells, 53BP1 recruitment acts as a barrier for HR by preventing DSBs’ end resection, however, BRCA1 is also expressed at this stage and can form IR-induced foci that colocalize with γH2AX foci in 53BP1 depleted cells [10]. Thus, we next aimed to determine whether 53BP1 deficient recruitment to DSBs in ADs would allow BRCA1 to ectopically form foci at DSB sites during G1. We first scored, in G1 cells (negative for CENPF staining), the γH2AX foci induced 30 min after localized DSB induction (Figure 2A, panel ii), and checked for BRCA1 foci colocalization inside the pore zone. Indeed, BRCA1/γH2AX foci colocalization was significantly higher in G1 cells from ADs (ADs: 41.66%; YDs: 17.41%; Mann–Whitney test; *p*-value < .0001) (Supplementary Table 4; Figure 4A, 4B). Moreover, the fraction of BRCA1-free γH2AX foci was lower in ADs (~10%) than in YDs (~40%) (Figure 4A), indicating that in G1 cells, BRCA1 can form foci at γH2AX-signaled DSBs and that the frequency of this event increases with the donor’s age. Since 53BP1 recruitment is compromised in ADs, it is tempting to speculate that ectopic BRCA1 recruitment is promoted at the 53BP1-orphan DSBs. In fact, the sum of the two colocalization frequencies (53BP1/γH2AX plus BRCA1/γH2AX) reaches ~100% in each donor (Figure 4B, 4C), suggesting that either 53BP1 or BRCA1 is recruited to almost all DSBs within 30 min of their induction. We propose that ectopic BRCA1 foci formation at DSBs in G1 cells is an age-related response, most probably related to an attempt to occupy DSBs that have failed to recruit 53BP1.

### CtIP-mediated DNA-end resection of DSBs occurs in G1 cells from aged donors

To further explore the functional relevance of the BRCA1 ectopic recruitment observed in G1 cells from ADs, we analyzed whether CtIP was also being recruited. To this end, HMECs from YDs and ADs were exposed to 5 Gy of γ-rays and the number of G1 cells positive for CtIP (≥ 5 foci) was scored. At 1 h pIR, ADs showed a significantly increased frequency of CtIP-positive cells (< 6% in YDs vs. > 15% in ADs; Fisher’s exact test; *p*-value < .05) (Figure 4D, 4E). These results suggest that, although not all of the γH2AX foci colocalizing with BRCA1 had efficiently recruited CtIP, a significant fraction of DSBs in AD cells eventually did and might undergo end resection.

Because RPA binds and stabilizes ssDNA intermediates that arise after DNA processing, we next analyzed its recruitment in G1 cells. At 1 h pIR, the number of RPA foci scored in G1 cells was negligible regardless of donor’s age (results not shown). We reasoned that an extensive resection that would allow RPA recruitment might require more time. At 4 h after irradiation most YD cells in G1 were still devoid of RPA foci, but the frequency of RPA-positive cells (≥ 5 foci) in AD cells had increased significantly (0% in YDs vs. ≥ 10% in ADs; Fisher’s exact test; *p*-value < .05) (Figure 4F, 4G). These results suggest that the initial DNA-end resection elicited by CtIP in G1 cells from older donors translates, with time, into an extensive DNA-end resection that results in single-stranded stretches of DNA that are effectively coated with RPA. Finally, the colocalization of RAD51 foci with γH2AX foci was extremely low in both young and aged donors at 4 h after 5 Gy exposure (2.12% in YDs vs. 2.47% in ADs; Kruskal–Wallis and Dunn’s multiple correction test; *p*-value > .05) (Figure 4H). Thus, DNA breaks occupied with BRCA1 in G1 cells from older donors are not processed by the HR pathway. However, the impact of those DSBs suffering DNA-end resection in G1 should be reckoned with, as DNA resected intermediates are able to suppress c-NHEJ-mediated DSB repair to favor alternative and highly mutagenic DNA repair mechanisms.

## DISCUSSION

The mechanisms by which older individuals accumulate genome rearrangements and DSBs have remained unknown for years. In a previous work, we reported a delayed firing of the DDR in G1 HMECs from aged donors that could contribute to the accumulation of DSBs with age [26]. In the present work, we explored the causes of the postponed ignition of the repair response in aged individuals. We observed a deficient recruitment of 53BP1 to radiation-induced DSBs from old donors, a defect that was previously described by our group using *in vitro* aged HMECs [37], suggesting that the deficiency in 53BP1 recruitment could be an age-related characteristic. Using the reporter plasmids technique, a decreased activity of the c-NHEJ in aged donors has also been detected. It has been described that 53BP1 acts with fast kinetics [13] and its rapid positioning at the break site along with its effector protein RIF1 impedes DNA-end resection and promotes c-NHEJ repair during G1 [10]. Therefore, the impaired recruitment of 53BP1 to DSBs described here could alter the triggering of DSB repair and could translate into defective c-NHEJ repair.

The deficient recruitment of 53BP1 to DSBs is not due to its depletion, as no age-associated differences were detected between the two age groups neither at mRNA nor at the protein level. We also discarded factors related to an aberrant DSB signaling. A defect in DSB detection would translate into an aberrant formation of γH2AX foci but, instead, results from our previous work demonstrated a higher frequency of γH2AX foci in cells from ADs [26]. However, aging is associated with changes at the epigenetic level [38], which could influence 53BP1 recruitment to DSBs. The accumulation of 53BP1 at DSB sites requires both recognition of H2AK15ub via its UDR motif and H4K20me2 via its Tudor domain [14, 33]. H2AK15ub marking is catalyzed by the RNF pathway that is also required for BRCA1 recruitment to DSBs [39]. Since BRCA1 showed no deficiency in foci formation at DSBs in HMECs, we discard that a failure in this pathway might be the cause of 53BP1 impaired recruitment in cells from aged women. Similarly, acetylation of H4K16 has been described to prevent the interaction of 53BP1 with H4K20me2 and consequent binding to DNA [17], but aged HMECs did not display hyperacetylation of this mark. In line with this, both aging and senescence processes have been linked to H4K16 hypoacetylation [40, 41]. Instead, we found an age-associated decreased expression of SETD8, the enzyme responsible for the monomethylation of H4K20. This result was previously reported in senescent and in *in vitro* aged cells [35, 36], demonstrating that SETD8 decreased expression is shared between different aging models. Decreased levels of methyltransferase SETD8 in HMECs from aged donors might result in the persistence of H4K20me0 sites in G1, compromising DSB recognition by 53BP1. In this regard, it has been recently demonstrated that the H4K20me0 mark is required for BRCA1 recruitment to post-replicative chromatin [19]. Thus, while 53BP1 recruitment to DSB at H4K20me0 sites would be hindered, BRCA1 recruitment would be enhanced.

Given the antagonistic role of 53BP1 and BRCA1 in the DSB repair pathway choice, it is not surprising that an impaired recruitment of 53BP1 would have consequences in BRCA1 dynamics. While the recruitment of BRCA1 was not affected by age in G2 cells, we observed an increased formation of ectopic BRCA1 foci in G1 cells from ADs. Ectopic BRCA1 and CtIP foci formation in G1 after inhibition or depletion of 53BP1 or its effector protein, RIF1, has been previously reported [10, 42]. In the present work 53BP1 is not depleted from ADs cells, but it is absent in as much as 40% of the radiation-induced DSBs, giving room to BRCA1 to occupy these breaks, most probably in an attempt to resolve the otherwise orphan DSBs. All of these results evidence the mutually exclusive relationship between BRCA1/CtIP and 53BP1/RIF1 tandems, which ultimately determine the DSB repair machinery that will be loaded onto the DSB. Thus, we propose that the age-associated repair defect in HMECs relies on the antagonism between 53BP1 and BRCA1 proteins that probably shift the DNA–DSB repair pathway choice to more mutagenic repair mechanisms.

The increased frequency of BRCA1 foci in G1 cells from ADs was concomitant with an increased frequency of CtIP foci. From S to G2 phases, CtIP activation is directly regulated by CDKs, but in G1 cells CtIP can be phosphorylated by polo-like kinase 3 (PLK3) [42, 43]. Additionally, not only CtIP, but also RPA foci formation in AD cells indicates that long stretches of ssDNA are generated in G1 in the absence of 53BP1, suggesting that extensive DNA-end resection has taken place in some DBSs in G1 cells. If CtIP initiates end resection in these breaks, c-NHEJ can no longer operate. Launching the HR in G1 cells is not possible due to the absence of a homologous repair template, together with BRCA1-PALB2-BRCA2 complex assembly inhibition by CDKs [44]. In agreement with this, RAD51 was not loaded in G1 cells from ADs, discarding the HR as an active repair pathway to process DNA-end resected DSBs in G1. We speculate that the age-associated ectopic activity of BRCA1/CtIP could account for the increased accumulation of genome rearrangements typically observed in aged tissues [2, 45]. CtIP has oncogenic properties due to its implication in the Alt-EJ pathway, and has been associated with chromosomal instability and the generation of aberrant chromosomal rearrangements [46].

While neither the c-NHEJ nor the HR operated in 53BP1-free DSBs in G1 cells from ADs, DNA breaks were actually being repaired, as only a small fraction of DSBs remain unrepaired a long time after irradiation [26]. It is tempting to speculate that these DSBs are being repaired by an alternative repair mechanism such as the Alt-EJ, which shares with HR the initial steps of DNA-end resection mediated by CtIP and MRE11, prior to the ligation of the DNA-ends [47]. The Alt-EJ can operate both in the G1 and G2 phases of the cell cycle [48] and in cells with functional c-NHEJ and HR pathways [47]. Contrary to c-NHEJ, Alt-EJ requires a greater degree of end resection prior to the DNA-end ligation step, often resulting in extensive losses, additions or alterations of the DNA sequence at the DSB junction. Also, the Alt-EJ functions with slower kinetics, thus increasing the probability of illegitimate repair [48]. All these scenarios are compatible with the reported increased frequencies of genome rearrangements and somatic mutations in tissues from aged individuals [2, 45].

**Figure 5:**
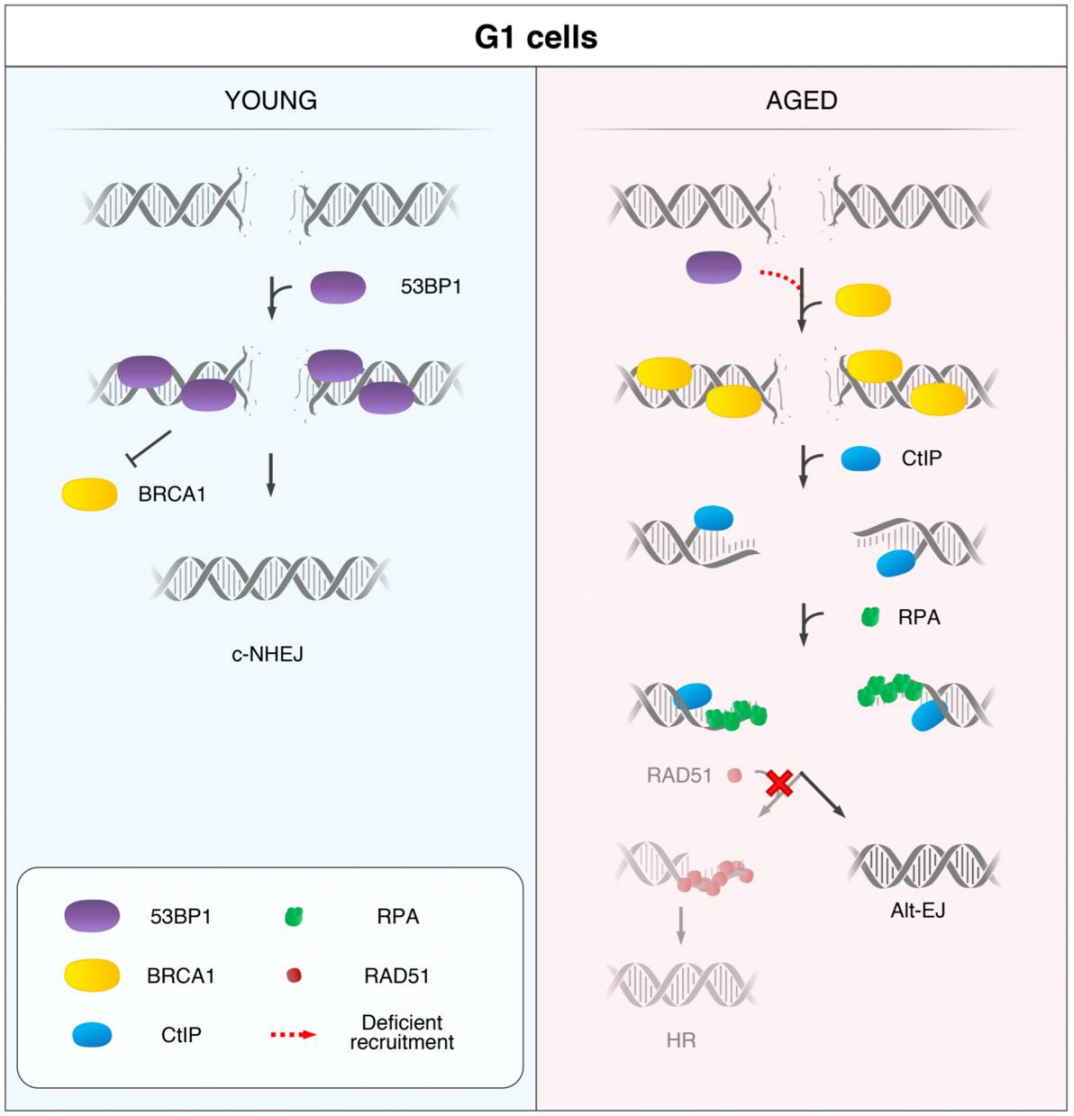
**Model for the age-related shift in the DSB repair pathway choice in G1 cells.** In response to DSB induction, 53BP1 is recruited to the break site in G1 cells from YDs and promotes repair by canonical non-homologous end-joining (c-NHEJ). Instead, in G1 AD cells, the deficient recruitment of 53BP1 permits the ectopic recruitment of BRCA1 to some DSBs, followed by CtIP-mediated DNA-end resection and RPA coating of the ssDNA. However, RAD51 loading is inhibited in these G1 cells and homologous recombination (HR) cannot be launched. Thus, DSBs from ADs that have suffered DNA-end resection in G1 become substrates for alternative DSB repair mechanisms, such as the alternative end-joining (Alt-EJ).

In summary, we propose that 53BP1 deficient recruitment to DSBs is a hallmark of age, and we present a model for a DSB repair pathway choice in HMECs from aged women (Figure 5). Upon induction of DSBs in YD cells, 53BP1 is recruited in the G1 phase and promotes c-NHEJ. In contrast, AD cells fail to recruit 53BP1 to DSBs efficiently, allowing their ectopic occupancy by BRCA1, which is followed by CtIP-mediated DNA-end resection. If resection is extensive enough, single-stranded fragments are coated by RPA while awaiting ligation. Because the c-NHEJ pathway is inhibited by extensive DNA-end resection of breaks and HR cannot be launched in G1, we speculate two possible outcomes for these DSBs: they might remain extensively processed and unrepaired until the cells progress to S phase and HR can be fully launched in the presence of a DNA template or, otherwise, they become substrates for alternative and highly mutagenic backup mechanisms of DNA repair, such as Alt-EJ repair.

The present work evidences the connection between genome integrity and aging. Future efforts addressing the exact mechanism to counteract BRCA1/CtIP activity during the G1 phase in cells from older individuals could be of extreme interest, as they would allow the restoration of DNA repair fidelity during aging and prevent a rise in genomic instability in older individuals.

## MATERIALS AND METHODS

### Cell culture

Finite lifespan pre-stasis HMECs were obtained from reduction mammoplasty of 11 donors: 48R (16 yo), 240L (19 yo), 168R (19 yo), 184 (21 yo), 59L (23 yo), 123 (27 yo), 153L (60 yo), 112R (61 yo), 122L (66 yo), 29 (68 yo) and 429ER (72 yo); and one peripheral non-tumor containing mastectomy tissue of one donor: 353P (72 yo). Donors were classified depending on their age into young donors (YDs ≤ 27 years) and aged donors (ADs ≥ 60 years). When referring to donors, the age group is followed by specimen identification and the age of the donor. HMECs were cultured as pre-stasis strains using M87A medium with cholera toxin and oxytocin according to previously reported methods [49], with the addition of 100 U/ml penicillin and 100 μg/ml streptomycin. U2OS cells were cultured with DMEM and Ham’s F10 medium at 1:1 and supplemented with 10% FBS and penicillin/streptomycin. Cells were kept in the incubator at 37° C and 5% CO_2_ atmosphere.

### Irradiation

Micro-localized DNA damage was induced as described by Suzuki et al. [27]. Briefly, cells were labelled with 10 μm bromodeoxyuridine (BrdU) for 48 h. Just before the irradiation medium was removed, cells were briefly rinsed with PBS and covered with a Whatman™ Cyclopore™ polycarbonate membrane (Whatman, Maidstone, UK) with pores of 5 μm in diameter. Cells were exposed to 15J m^-2^ of UVC light at a dose rate of 1J m^-2^ s^-1^. After exposure, free-BrdU medium was added and cells were incubated at 37° C and 5% CO_2_ for 15–60 min. Whole-cell irradiation was performed by exposing cells to 5 Gy of γ-rays using an IBL-437C R-137 Cs irradiator at a dose rate of 5.10 Gy/min.

### Immunofluorescence

HMECs were fixed during 15 min with 4% PFA, permeabilized with 1x PBS-0.5% Triton-X-100 solution for 20 min and blocked with 1x PBS – 0.5% BSA – 0.15% glycine for 15 min. For RAD51 and CtIP labelling, cells were also fixed with ice-cold methanol for 30 min after PFA, permeabilized with ice-cold acetone for 1 min and blocked with 1x PBS – 1% FBS – 5% BSA. For H4K16ac labeling, after permeabilization with triton, cells were incubated for 1 h at 60° C with Dako REAL^TM^ Target Retrieval Solution (Agilent Technologies, Santa Clara, CA, USA). After the blocking step, primary antibodies (listed in Supplementary Table 5) were incubated overnight at 4° C. After three washes with 1x PBS –0.1% Tween20 or PBS – 1% FBS, the secondary antibodies anti-rabbit A488 and anti-mouse A594 (Supplementary Table 5) were incubated for 1 h at room temperature. If a third protein was detected (CENPF), secondary antibodies from the other two were reincubated in order to occupy the maximum number of epitopes before anti-CENPF was incubated overnight at 4° C. The secondary antibody for CENPF was then incubated for 20 min at room temperature. Finally, all samples were washed, briefly rinsed with distilled water, underwent progressive alcohol dehydration and counterstained with 4’,6-diamidino-2-phenylindole (DAPI) at a final concentration of 0.25 μg/ml in Vectashield Mounting Medium (Vector Laboratories, Inc., Burlingame, CA, USA). Analysis and image acquisition were performed using an Olympus BX61 epifluorescent microscope (Olympus, Hamburg, Germany) equipped with a CV-M4+CL camera (JAI, Grosswallstadt, Germany) and Cytovision software (Applied Imaging, Newcastle, UK). Fiji software [50] was used for H4K16ac fluorescence intensity measurement.

### Western blot

HMECs were washed with cold PBS and stored at -80° C upon collection. Cells were lysed in RIPA buffer (50 mM Tris-HCl pH 8, 1% sodium deoxycholate, 150 mM sodium chloride, 20 mM sodium fluoride, 1% Triton-X100, 1 mM EDTA, 0,1% SDS, DTT 1 mM, 20 mM b-glicerolphosphate, 1 mM Na ortovanadate and protease inhibitor cocktail (Roche, Basel, Switzerland) and sonicated. The protein concentration was measured using a Pierce^TM^ BCA Protein Assay Kit (Thermo Fisher Scientific, Waltham, MA, USA) and 25 μg of total protein was loaded onto a 7.5% acrylamide TGX Stain-Free gel or a 10% Bis-Tris gel. After electrophoresis, proteins were transferred to a PVDF membrane. After a blocking step with 5% BSA or 5% nonfat milk for 1 h, primary antibodies (Supplementary Table 5) were incubated overnight at 4° C. After three washing steps with TTBS (Tris 20 mM, NaCl 150 mM, and 0.1% Tween20), secondary antibodies (Supplementary Table 5) were incubated for 1 h at room temperature. Chemiluminescent detection was performed after incubation with Immobilon Western Chemiluminescent HRP Substrate (Millipore, Madrid, Spain) and using a ChemiDoc^TM^ Touch Imaging System (Bio-Rad, Hercules, CA, USA). For reprobing, a mild stripping solution (1.5% glycine, 0.1% SDS, 1% Tween20) adjusted to pH 2.2 was used for antibodies with the same host species. An incubation in 30% H_2_0_2_ was applied for membrane reprobing with antibodies with different host species.

### Transfection with DNA repair reporter plasmids and flow cytometric analysis

To measure c-NHEJ and HR repair activity, 40,000 cells from an exponentially growing culture were seeded onto 12-well plates. When properly attached, cells were transfected with 0.5 μg of reporter pimEJ5GFP (Addgene #44026) or pDRGFP (Addgene #26475) and with 0.5 μg of the I-SceI-expressing plasmid pCBASceI (Addgene #26477) or with an empty vector pCAGGS (kind gift from Surrallés’ and Jasin’s laboratories) as a negative control. As a positive control, cells were transfected with 0.5 μg of the GFP-expressing plasmid NZE-GFP (kind gift from Surrallés’ and Jasin’s laboratories). The mix of DNA was prepared in OptiMEM medium and then mixed in a 1:4 proportion with Fugene® HD transfection reagent (Promega, Madison, WI, USA) following the manufacturer’s instructions. Transfection mix was added to cells in 1 ml of antibiotic-free medium and incubated for 13 h at 37° C and 5% CO_2_ atmosphere. The percentage of GFP-positive cells was measured by flow cytometry with a FACS Calibur cytometer (Becton Dickinson, Franklin Lakes, NJ, USA) at 40 h after transfection. FlowJo software (v10.0.7; FlowJo LLC, Ashland, OR, USA) was used for gate adjusting and plot representation. The threshold of GFP-negative cells was determined for each donor with values from cells transfected with the pCAGGS empty vector. To compare activity levels of each repair pathway between donors, the fluorescent value obtained for each reporter plasmid was normalized with the transfection efficiency value obtained after transfection with the GFP-expressing plasmid NZE-GFP. At least two independent transfections with the reporter plasmids and their respective controls were performed for each donor.

### Cell cycle by flow cytometry

A cell cycle analysis was performed as described elsewhere [51]. Briefly, after trypsinization, cells were fixed in ethanol and stored at -20° C. After all samples were collected, cells were stained with PI staining solution (0.1% Triton-X-100 in PBS, 0.2 mg/ml DNase-free RNase A and 0.02 mg/ml of propidium iodide). After 30 min of incubation at room temperature, fluorescence intensity was measured using a FACS Calibur cytometer (Becton Dickinson) and analyzed using FlowJo software.

### RNA isolation, cDNA synthesis and quantitative real-time PCR

For RNA collection, cells were lysed using TRIzol^TM^ solution (Thermo Fisher Scientific, Waltham, MA, USA) and stored at -80° C until all samples were collected. After thawing, chloroform was added at 1:5 (v/v), incubated for 2 minutes and centrifuged at 12000 g for 15 minutes at 4° C. RNA was purified from the resulting aqueous phase using Maxwell® RSC simplyRNA kit (Promega, Madison, WI, USA) and following manufacturer’s instructions. RNA quantity and purity were measured using NanoDrop^TM^ 2000 spectrophotometer (Thermo Fisher Scientific, Waltham, MA, USA). Samples scored purity odds ratios 260/280 and 260/230 between 1.9 and 2.1. RNA integrity was measured with an Agilent 2100 Bioanalyzer (Agilent Technologies, Santa Clara, CA, USA) and it reached the maximum score (RIN = 10). Retrotranscription of 1 μg of total RNA was performed using iScript cDNA synthesis kit (Bio-Rad, Hercules, CA, USA) in a final volume of 20 μL. Real-time quantitative PCR was performed using SYBR® Green (Bio-Rad, Hercules, CA, USA) on a CFX384 thermal cycler with CFX Manager software (Bio-Rad, Hercules, CA, USA). For each reaction, 50 ng of cDNA were used and samples were run in triplicates. SETD8 and ACTB primer sequences were obtained from the literature [36, 52]. Otherwise, 53BP1 and GAPDH were designed using the Primer3 online tool [53]. All primers were purchased from Condalab (Metabion, Munich, Germany) and are listed in Supplementary Table 6. Data normalization was performed using the geometric mean of GAPDH and ACTB reference genes. Fold change was calculated following the 2^-ΔΔCt^ method [54].

### Statistical analysis

The data obtained were analyzed using Microsoft Excel (Microsoft® Excel® 2011, v14.1, Redmond, Washington, USA). The statistical analysis was performed using GraphPad Prism 8 (GraphPad Software Inc., San Diego, CA, USA) with methods indicated in the results where applicable. For the hierarchical cluster analysis, the Ward method was applied [55] using R software (version 3.4.4, Vienna, Austria).

## Supplementary Material

Supplementary Figures

Supplementary Tables
