# Supplementary material for: Age-associated deficient recruitment of 53BP1 in G1 cells directs DNA double-strand break repair to BRCA1/CtIP-mediated DNA-end resection
Source: Aging (Albany NY). 2020 Dec 27;12(24):24872–93. doi: 10.18632/aging.202419 (PMC7803562; doi:10.18632/aging.202419)
Supplement: Supplementary Figures [file aging-12-202419-s001.pdf]

SUPPLEMENTARY FIGURES

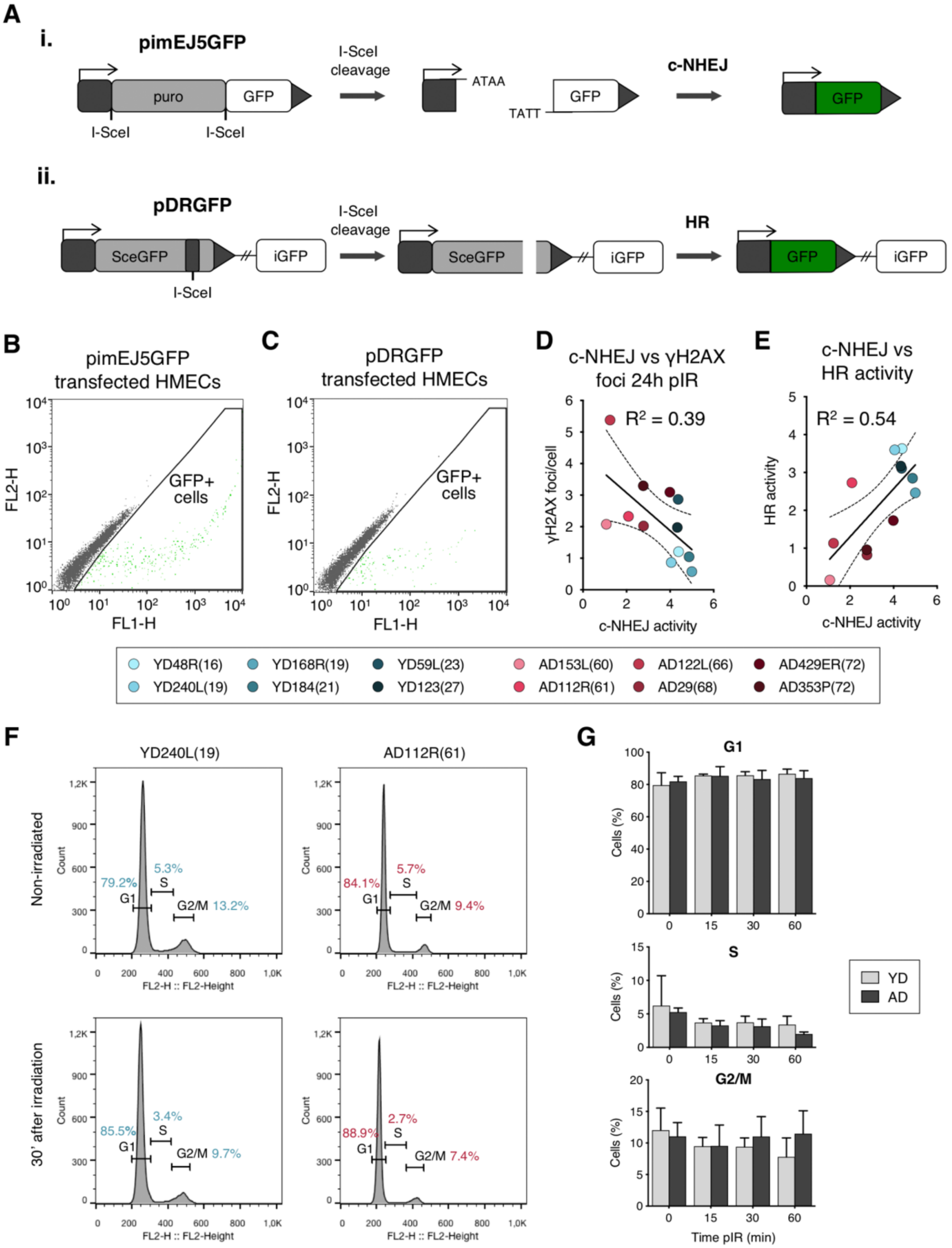

Supplementary Figure 1. Reporter plasmids assays and cell cycle analysis with flow cytometry. (**A**) Structure of the reporter plasmids for the analysis of (i) c-NHEJ and (ii) HR efficiency. (**B**, **C**) Representative flow cytometry plots of the frequency of GFP-positive cells after co-transfection with the c-NHEJ reporter plasmid pimEJ5GFP (**B**) or the HR reporter plasmid pDRGFP (**C**) and the I-SceI expressing plasmids. (**D**, **E**) Correlation between c-NHEJ activity and the residual number of γH2AX foci (**D**) or between c-NHEJ and HR pathways' activity

(**E**) in HMECs from YDs and ADs. Best-fit line, 95% confidence bands (dotted lines) and Pearson's correlation coefficient (R^2) are indicated (p < .05). (**F**) Representative diagrams of cell cycle distribution for YDs and ADs before and at 30 min post-irradiation (15 J/m², UVC, BrdU sensitized cells). (**G**) Frequency of cells at G1, S and G2/M stages in non-irradiated HMECs and at 15, 30 and 60 min after irradiation (15 J/m², UVC, BrdU sensitized cells). Mean values and SD from three young donors (YD240L(19), YD168R(19) and YD184(21)) and three ADs (AD112R(61), AD122L(66) and AD429ER(72)) are shown.

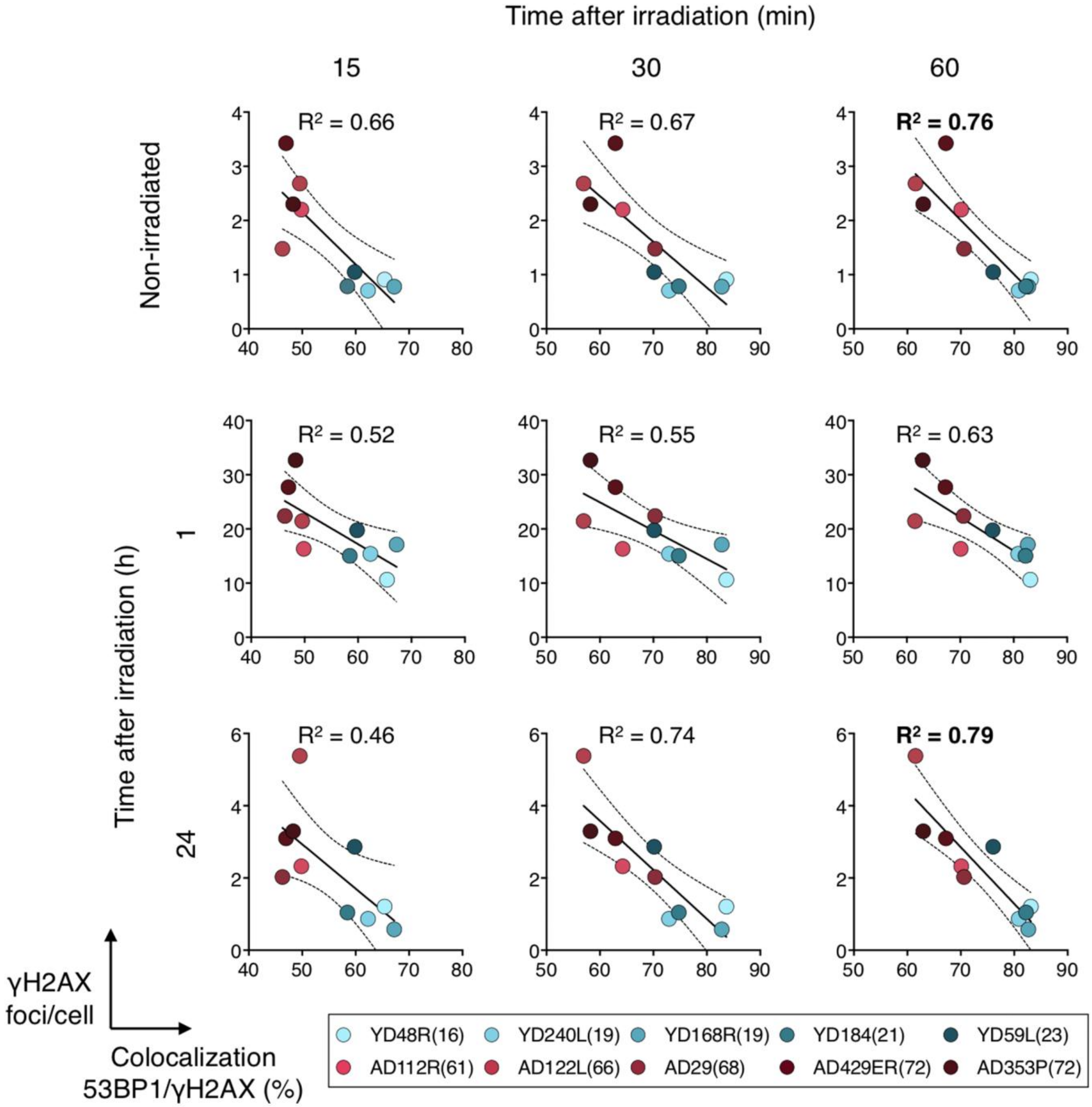

Supplementary Figure 2. Negative correlations between 53BP1/γH2AX foci colocalization and the number of DSBs scored as γH2AX foci in HMECs from YDs and ADs. 53BP1/γH2AX foci colocalization was evaluated at 15, 30 and 60 min pIR. γH2AX foci were scored before irradiation and 1 h and 24 h pIR. Best-fit line, 95% confidence bands (dotted lines) and Pearson's correlation coefficient (R^2) are indicated (p < .05). Most significant values are highlighted in bold letters.
