# Supplementary material for: Age-associated deficient recruitment of 53BP1 in G1 cells directs DNA double-strand break repair to BRCA1/CtIP-mediated DNA-end resection
Source: Aging (Albany NY). 2020 Dec 27;12(24):24872–93. doi: 10.18632/aging.202419 (PMC7803562; doi:10.18632/aging.202419)
Supplement: Supplementary Tables [file aging-12-202419-s002.pdf]

SUPPLEMENTARY TABLES

Supplementary Table 1. Plasmid reporter assays for the c-NHEJ and HR pathways in HMECs.

Donor	Transfection efficiency		c-NHEJ	HR
	%	SD	Normalized %	Normalized %
**Young donors**	**9.18**	**5.75**	**4.50**	**3.14**
YD48R(16)	10.67	0.40	4.39	3.63
YD240L(19)	4.21	1.01	4.05	3.60
YD168R(19)	11.94	4.40	5.00	2.46
YD184(21)	5.51	1.94	4.87	2.85
YD59L(23)	8.11	0.43	4.37	3.11
YD123(27)	22.35	0.78	4.33	3.17
**Aged donors**	**11.51**	**7.74**	**2.32**	**1.33**
AD153L(60)	22.03	3.31	1.07	0.16
AD112R(61)	16.62	9.45	2.09	2.73
AD122L(66)	4.99	0.79	1.24	1.13
AD29(68)	6.16	0.60	2.78	0.82
AD429ER(72)	7.51	5.73	3.99	1.73
AD353P(72)	11.93	1.56	2.77	0.96

Supplementary Table 2. Colocalization of 53BP1 and γH2AX foci at radiation-induced DSBs in HMECs.

Donor	15' pIR				30' pIR				60' pIR			
	N pores	N γH2AX foci	N 53BP1 foci	% 53BP1/ γH2AX	N pores	N γH2AX foci	N 53BP1 foci	% 53BP1/ γH2AX	N pores	N γH2AX foci	N 53BP1 foci	% 53BP1/ γH2AX
**Young donors**	**440**	**4250**	**2660**	**62.59**	**451**	**4193**	**3203**	**76.39**	**453**	**4227**	**3424**	**81.00**
YD48R(16)	56	486	318	65.43	54	361	302	83.66	62	527	438	83.11
YD240L(19)	110	1245	776	62.33	108	1086	792	72.93	107	1117	903	80.84
YD168R(19)	109	988	664	67.21	110	1121	928	82.78	104	1061	877	82.66
YD184(21)	107	1023	598	58.46	92	895	669	74.75	94	787	647	82.21
YD59L(23)	58	508	304	59.84	87	730	512	70.14	86	735	559	76.05
**Aged donors**	**467**	**4692**	**2261**	**48.19**	**464**	**4510**	**2815**	**62.42**	**411**	**3701**	**2472**	**66.79**
AD112R(61)	106	1025	511	49.85	103	1035	665	64.25	105	933	654	70.10
AD122L(66)	105	1126	558	49.56	109	1189	677	56.94	93	894	550	61.52
AD29(68)	110	1242	575	46.30	109	1021	718	70.32	98	878	620	70.62
AD429ER(72)	92	796	374	46.98	50	396	249	62.88	64	491	330	67.21
AD353P(72)	54	503	243	48.31	93	869	506	58.23	51	505	318	62.97

Supplementary Table 3. Colocalization of BRCA1 and γH2AX foci at radiation-induced DSBs in CENPF-positive (G2) HMECs.

Donor	15' pIR				30' pIR				60' pIR			
	N pores	N γH2AX foci	N BRCA1 foci	% BRCA1/ γH2AX	N pores	N γH2AX foci	N BRCA1 foci	% BRCA1/ γH2AX	N pores	N γH2AX foci	N BRCA1 foci	% BRCA1/ γH2AX
**Young donors**	**94**	**1107**	**471**	**42.55**	**86**	**1050**	**685**	**62.67**	**83**	**1231**	**830**	**67.42**
YD48R(16)	15	108	45	41.67	16	124	81	65.32	8	74	51	68.92
YD240L(19)	28	407	178	43.73	31	460	284	61.74	30	549	376	68.49
YD168R(19)	22	256	109	42.58	18	211	135	63.98	25	367	243	66.21
YD184(21)	29	336	139	41.37	21	255	158	61.96	20	241	160	66.39
**Aged donors**	**87**	**1034**	**445**	**43.04**	**93**	**1238**	**766**	**61.87**	**106**	**1439**	**933**	**64.84**
AD112R(61)	28	340	139	40.88	23	321	202	62.93	32	405	255	62.96
AD29(68)	24	300	131	43.67	26	359	217	60.45	31	425	284	66.82
AD429ER(72)	26	245	108	44.08	30	373	237	63.54	29	389	250	64.27
AD353P(72)	9	149	67	44.97	14	185	110	59.46	14	220	144	65.45

Supplementary Table 4. Colocalization of BRCA1 and γH2AX foci at radiation-induced DSBs in CENPF-negative (G1) HMECs.

Donor	30' pIR			
	N pores	N γH2AX foci	N BRCA1 foci	% BRCA1/ γH2AX
**Young donors**	**90**	**896**	**156**	**17.41**
YD48R(16)	31	261	46	17.62
YD240L(19)	29	351	63	17.95
YD168R(19)	30	284	47	16.55
**Aged donors**	**86**	**761**	**317**	**41.66**
AD112R(61)	27	268	95	35.45
AD122L(66)	26	203	100	49.26
AD429ER(72)	33	290	122	42.07

Supplementary Table 5. List of antibodies used.

Antibody	Host	Reference	Working dilution	
			IF	WB
**Primary antibodies**				
Anti-53BP1	Rabbit	Abcam, ab21083	1:2000	1:1000
Anti-BRCA1	Mouse	Abcam, ab16781, clone MS13	1:500	1:500
Anti-CENPF	Rabbit	Abcam, ab90, clone 14C10/1D8	1:1000	-
Anti-CtIP	Mouse	Millipore, MABE1060, clone 14-1	1:500	-
Anti-Histone H4K16ac	Rabbit	Active Motif, 39930	1:200	-
Anti-Integrin β1	Rabbit	Abcam, ab52971	-	1:1000
Anti-Ku70	Rabbit	Abcam, ab83501	-	1:3000
Anti-Pericentrin	Rabbit	Abcam, ab4448	1:2000	-
Anti-RAD51	Rabbit	Abcam, ab63801	1:15000	1:2000
Anti-RPA32/RPA2	Mouse	Abcam, ab2175, clone 9H8	1:500	1:1000
Anti-γH2AX (Ser139)	Mouse	Millipore, 05-636, clone JBW301	1:1000	-
Anti-γH2AX (Ser139)	Rabbit	Abcam, ab81299, clone EP854(2)Y	1:500	-
**Secondary antibodies**				
Anti-mouse HRP conjugate	Goat	Millipore, 12-349	-	1:5000
Anti-mouse Alexa Fluor® 488	Goat	Jackson ImmunoResearch Inc., 115-545-205	1:500	-
Anti-mouse Cyanine Cy™3	Goat	Jackson ImmunoResearch Inc., 115-165-146	1:800	-
Anti-rabbit HRP conjugate	Goat	Millipore, 12-384	-	1:5000
Anti-rabbit Alexa Fluor® 488	Goat	Thermo Fisher Scientific, A-11034	1:500	-
Anti-rabbit Alexa Fluor® 532	Goat	Thermo Fisher Scientific, A-11009	1:1000	-
Anti-rabbit Alexa Fluor® 594	Goat	Thermo Fisher Scientific, A-11037	1:500	-

Supplementary Table 6. Sequences of RT-qPCR primers.

Target	Forward primer	Reverse primer
53BP1	5'-TTCCTCAACATCCTGGCTCT-3'	5'-ACATTCCCTTGGTGCTGAAG-3'
ACTB	5'-CCAACCGCGAGAAGATGA-3'	5'-CCAGAGGCGTACAGGGATAG-3'
GAPDH	5'-AGCCACATCGCTCAGACAC-3'	5'-GCCCAATACGACCAAATCC-3'
SETD8	5'-TCTTGTGATTCCACCAATGC-3'	5'-GGACAGGGTAGAAATCCGT-3'
